# Dependence of Nociceptive Detection Thresholds on Physiological Parameters and Capsaicin-Induced Neuroplasticity: A Computational Study

**DOI:** 10.3389/fncom.2016.00049

**Published:** 2016-05-25

**Authors:** Huan Yang, Hil G. E. Meijer, Robert J. Doll, Jan R. Buitenweg, Stephan A. van Gils

**Affiliations:** ^1^Applied Analysis, MIRA Institute for Technical Medicine and Biomedical Technology, University of TwenteEnschede, Netherlands; ^2^Biomedical Signals and Systems, MIRA Institute for Technical Medicine and Biomedical Technology, University of TwenteEnschede, Netherlands

**Keywords:** nociceptive detection, detection threshold, capsaicin, neuroplasticity, computational modeling

## Abstract

Physiological properties of peripheral and central nociceptive subsystems can be altered over time due to medical interventions. The effective change for the whole nociceptive system can be reflected in changes of psychophysical characteristics, e.g., detection thresholds. However, it is challenging to separate contributions of distinct altered mechanisms with measurements of thresholds only. Here, we aim to understand how these alterations affect Aδ-fiber-mediated nociceptive detection of electrocutaneous stimuli. First, with a neurophysiology-based model, we study the effects of single-model parameters on detection thresholds. Second, we derive an expression of model parameters determining the functional relationship between detection thresholds and the interpulse interval for double-pulse stimuli. Third, in a case study with topical capsaicin treatment, we translate neuroplasticity into plausible changes of model parameters. Model simulations qualitatively agree with changes in experimental detection thresholds. The simulations with individual forms of neuroplasticity confirm that nerve degeneration is the dominant mechanism for capsaicin-induced increases in detection thresholds. In addition, our study suggests that capsaicin-induced central plasticity may last at least 1 month.

## 1. Introduction

The nociceptive system processes pain-related information and its function results from the delicate balance of a myriad of mechanisms (Coutaux et al., 2005; Latremoliere and Woolf, 2009; Sandkühler, 2009). Clinical interventions can perturb or recover this balance in multiple ways (Kyranou and Puntillo, 2012). Changes in the whole nociceptive system may be assessed with quantitative sensory testing (Wilder-Smith, 2002; Maier et al., 2010). However, separate contributions of distinct mechanisms are difficult to assess, which may be unraveled partially by using a modeling approach accounting for the relevant mechanisms.

The nociceptive system contains multiple pathways to detect noxious stimuli, i.e., actually or potentially tissue damaging events. The peripheral paths start with myelinated Aδ- and non-myelinated C-fibers, e.g., with nerve endings in the skin, responding to such stimuli. These fibers project to secondary neurons in the dorsal horn. Central terminals of both types of fibers contact secondary neurons extensively within the superficial dorsal horn, where nociceptive information is relayed to supra-spinal structures (Todd, 2010). Changes in the neurophysiological properties of both peripheral and central nociceptive subsystems can be induced by diseases, clinical interventions, or experimental conditioning stimuli, and can result in sensitization manifesting itself as hyperalgesia (Sandkühler, 2009). Sensitization is hypothesized to be one important factor leading to persistent pain (Latremoliere and Woolf, 2009; Woolf, 2011). For an improved treatment of persistent pain, it is important to understand the individual and combined contributions of peripheral and central nociceptive processing.

To study neuroplasticity underlying sensitization processes, we need to preferentially measure activation of nociceptive subsystems. Electrocutaneous stimulation at low stimulus amplitudes achieves this preferential activation of Aδ-fibers (Inui et al., 2002; Mouraux et al., 2010; Mørch et al., 2011; Steenbergen et al., 2012). Furthermore, electrical stimulation can be applied as a square wave pulse train characterized by three temporal properties, namely the number of pulses (*NoP*), the interpulse interval (*IPI*), and the pulse width (*PW*). The relation between the detection probability and stimulus amplitudes can be studied with fixed temporal properties. This relation, referred to as the psychometric function, is different for various values of temporal properties. A short *PW* (i.e., < 1 ms) controls the activation of Aδ fibers, whereas the *IPI* is in the order of tens of milliseconds and contributes to temporal summation of neuronal activity of dorsal horn neurons. To study nociceptive processing underlying the detection task, we developed a computational model accounting for activation of Aδ-fibers and central processing by secondary dorsal horn neurons (Yang et al., 2015). This computational model is consistent with the principle of probability summation in psychophysical studies, and we refer to it as the hazard model (HM). The computational convenience of the HM may facilitate further model-based studies. In addition, the model replicated the experimentally observed dependence of detection thresholds on temporal stimulus properties for healthy subjects. However, it was not addressed how changes of parameters due to neuroplasticity affect detection thresholds.

**Figure 1:**
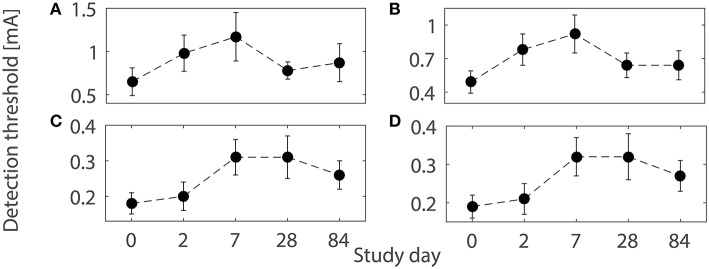
**Detection thresholds (mean ± SEM) using the four combinations of temporal properties on five study days before and after 1-h capsaicin treatment (reproduced from Doll et al., 2016b under the terms of the Creative Commons Attribution License)**. **(A)** (*NoP* = 1, *PW* = 0.21 ms), **(B)** (*NoP* = 1, *PW* = 0.525 ms), **(C)** (*NoP* = 2, *IPI* = 20 ms, *PW* = 0.525 ms), and **(D)** (*NoP* = 2, *IPI* = 50 ms, *PW* = 0.525 ms), respectively.

As a tool in pain research and as a therapeutic agent, capsaicin, the pungent substance in chili peppers, is widely used due to its capability to induce multiple forms of plasticity (O'Neill et al., 2012). Topical application of capsaicin is used as an experimental human pain model, thereby altering normal nociceptive functioning (Schmelz and Kress, 1996; Petersen and Rowbotham, 1999) accompanied by changes in peripheral and central nociceptive properties. Topical application of capsaicin includes activation of afferent fibers via the transient receptor potential vanilloid 1 (TRPV1) by increasing calcium and sodium influx into peripheral fibers (O'Neill et al., 2012). A high level of intracellular calcium ions causes the degeneration of nerve endings. In addition to structural changes, activation of C-fibers triggers the release of neuropeptides leading to sensitization of peripheral nerve endings and dorsal horn neurons (Coutaux et al., 2005; Todd, 2010). A significant effect of 8%-topical capsaicin treatment on detection thresholds was observed over three months in a human subject study for healthy subjects (Doll et al., 2016b). In addition, different patterns of changes of detection thresholds for single- and double-pulse stimuli were observed, shown in Figure 1. However, a mechanistic explanation is not straightforward as changes in multiple subsystems may be involved.

Here, we propose and demonstrate a model-based approach to understand the effects of neuroplasticity on nociceptive detection of electrocutaneous stimuli. With the hazard model, we study how variations in single-model parameters affect detection thresholds by parameter sweeping. Next, we find that the dependence of detection thresholds on the *IPI* for double-pulse stimuli can be non-monotone for which we derive an explicit condition. As a case study using 8%-topical capsaicin treatment, we translate neuroplasticity into plausible perturbations of model parameters over three months. We compare patterns of changes in model-based detection thresholds with experimentally obtained patterns qualitatively. We discuss how our findings could be used for future experiments or further exploration of capsaicin-induced effects relevant to pain management.

## 2. Materials and methods

We begin with a brief description of a previously built computational model of Aδ-fiber-mediated detection of electrocutaneous stimuli. Next, to understand possible neuroplasticity, we vary each model parameter to study its effects on detection thresholds. For double-pulse stimuli, we derive a quantity determining the monotonicity of detection thresholds with respect to the interpulse interval. Lastly, to investigate capsaicin-induced neuroplasticity, we simulate our model with plausible perturbations of model parameters.

### 2.1. Computational modeling of nociceptive detection of electrocutaneous stimulation

In psychophysical experiments, electrocutaneous stimuli are delivered by an intra-epidermal needle electrode (Steenbergen et al., 2012; Doll et al., 2014). In this experiment, stimuli with multiple combinations of temporal properties (*NoP*, *IPI*, and *PW*) were applied to a human subject. The inter-stimulus interval was 3 s on average. The experimental details were reported in Doll et al. (2016a,b). For each stimulus, the binary response *R* was recorded, i.e., *R* = 1 if the stimulus was detected, and *R* = 0 if not. Fixing the three temporal properties, a psychometric function describes the conditional probability to detect a stimulus with the amplitude (*A*), i.e., *Pr*(*R* = 1|*A*). In our modeling study, we denote Ψ(*A*) as the model-based psychometric function. The binary response in a single trial can be simulated by drawing a random number ξ from a standard uniform distribution. The response is *R* = 1 when ξ < Ψ(*A*), indicating that the stimulus is detected, and *R* = 0 otherwise. We denote the model-based detection threshold by *A*_50_, and it is implicitly defined by Ψ(*A*_50_) = 0.5. As Ψ(*A*) is an increasing function of *A*, the threshold *A*_50_ is well defined if Ψ(0) < 0.5. Then, *A*_50_ can be determined numerically.

Here, we briefly describe the model that represents essential mechanisms of Aδ-fiber-mediated nociceptive processing (Yang et al., 2015). We start with peripheral mechanisms. Electrocutaneous stimulation at low amplitudes focally recruits nerve endings of Aδ fibers (Mouraux et al., 2010). According to the cutaneous innervation (Provitera et al., 2007; Granstein and Luger, 2009), one can treat nerve endings perpendicular to the skin surface, where the needle electrode was attached. Upon brief stimulation (i.e., *PW* < 1 ms), peripheral activation depends on both the geometry (e.g., the depth of nerve endings *h*) and neurophysiological properties of both endings (e.g., the time constant τ_1_ and the firing threshold *V*_*th*_) and the skin (e.g., electrical conductivity). Hence, peripheral activation is described by the threshold-linear function [_*f*_*A*_ − α_1_]+_: = π(*f*_*A*_ − α_1_)*H*(*f*_*A*_ − α_1_), where $f_{A}:=A\left(1-\exp\left(-\frac{PW}{\tau_{1}}\right)\right)$, *H*(·) is the Heaviside step function, and α_1_ is the activation threshold of afferent fibers. In the model, electrical conductivity and resistance of the skin are absorbed in the lumped parameter α_1_, see its expression in Table 1.

**Table 1:** **Summary of physical and lumped parameters in the computational model**.

**PHYSICAL QUANTITIES**					
**Symbol**	**Description**				
*V*_*th*_	Firing threshold of afferent fibers				
*c*_0_	Conductivity of the superficial tissue under skin				
*c*_1_	Electrical resistance of nerve endings per unit length				
*C*_1_	Membrane capacitance of nerve endings				
*G*_1_	Membrane conductance of nerve endings				
ρ	Surface density of nerve endings				
*h*	Distance between nerve endings and the electrode				
ḡ	Maximal conductance of the AMPA-mediated synapse				
*K*	Potential gradient between the postsynaptic potential and the AMPA-reversal potential				
*C*_2_	Membrane capacitance of secondary neurons				
*G*_2_	Membrane conductance of secondary neurons				
α_*h*_	Activation threshold of secondary neurons				
σ_*h*_	Slope parameter of the activation of secondary neurons				
*l*	Number of secondary neurons				
λ_*h*_	Maximal firing rate of single secondary neurons				
**LUMPED PARAMETERS**					
**Symbol**	**Description**		**Expression**		**Reference value**
α_1_	Activation threshold of afferent fibers		$\left(4\pi c_{0}c_{1}G_{1}V_{th}\right)h^{2}$		0.125 mA
τ_1_	Time constant of afferent fibers		$C_{1}G_{1}^{-1}$		0.2 ms
τ_2_	Time constant of secondary neurons		$C_{2}G_{2}^{-1}$		45 ms
α_*L*_	Activation threshold of secondary neurons		$\left(\frac{8}{3}\pi c_{0}c_{1}G_{1}G_{2}V_{th}\right)\alpha_{h}{\left(\rho ḡK\right)}^{-1}$		0.00417 A/s
σ_*L*_	Slope parameter of firing rate function		$\left(\frac{8}{3}\pi c_{0}c_{1}G_{1}G_{2}V_{th}\right)\sigma_{h}{\left(\rho ḡK\right)}^{-1}$		8.33 × 10^−5^ A/s
λ_*L*_	The maximal population firing rate		*lλ*_*h*_		0.01 kHz

As Aδ-fibers are myelinated, the evoked spikes propagate robustly along the axons. Along the way to synapse onto a secondary neuron, the T-junction of a dorsal root ganglion can act as a low-pass filter if the frequency of the spike train is high, resulting in propagation failures (Stoney, 1990; Zhou and Chiu, 2001). As the time between consecutive stimuli is only a few seconds, the evoked spike train has a relatively low frequency. In addition, the experimentally used *PW* is relatively short (i.e., < 1 ms) (Doll et al., 2016a,b). Hence, it is unlikely to have multiple spikes evoked by a single pulse. Therefore, the model does not include propagation processes.

Next, through synaptic connections, an excitatory postsynaptic current (PSC) $I_{p}^{*}\left(t\right)$ is given as:

(1) $$\begin{array}{l} I_{p}^{*}\left(t\right)=\frac{{\left[f_{A}-\alpha_{1}\right]}_{+}}{\tau_{s}}\overset{NoP-1}{\underset{k\;=\;0}{\sum }}\exp\left(-\frac{t-kIPI}{\tau_{s}}\right)H\left(t-kIPI\right), \end{array}$$

with time constant τ_*s*_ = 1.5 ms (Gabbiani et al., 1994). There are more mechanisms, such as short-term synaptic plasticity, that affect PSCs, but both facilitation and depression have been reported (Luo et al., 2014). As the overall effect is unclear, we do not incorporate this in the model. The PSC drives the postsynaptic potential (PSP) of a secondary neuron in the dorsal horn *x*(*t*), which we model as a leaky integrator,

(2) $$\begin{array}{l} \tau_{2}ẋ=-x+I_{p}^{*}\left(t\right),\;x\left(0\right)=0. \end{array}$$

The time constant τ_2_ has a value of about several tens of milliseconds (Prescott and Koninck, 2002; Weng et al., 2006). The spike generation of a secondary neuron depends on the PSP together with other factors, which act as noise. Therefore, we model its spiking behavior as a non-homogeneous Poisson process with an instantaneous firing rate (Plesser and Gerstner, 2000) given by:

(3) $$\begin{array}{l} \lambda \left(t\right)=\lambda_{L}{\left(1+\exp\left(\frac{\alpha_{L}-x\left(t\right)}{\sigma_{L}}\right)\right)}^{-1}. \end{array}$$

Here, the lumped parameters α_*L*_, σ_*L*_, and λ_*L*_ represent the threshold, the slope parameter, and the maximal firing rate, respectively. The expected value of the number of spikes during a trial with the interval of duration *T* is

(4) $$\begin{array}{l} \lambda_{T}=\int_{0}^{T}\lambda \left(t\right)dt. \end{array}$$

This model assumes that the binary response *R* equals one if at least one secondary neuron generates an action potential during the trial interval *T* (Yang et al., 2015). Hence, the model-based psychometric function is given by:

(5) $$\begin{array}{l} \text{Ψ}=1-\exp\left(-\lambda_{T}\right). \end{array}$$

Our model contains six lumped parameters, which depend on more than 10 physical quantities, characterizing peripheral, and central nociceptive subsystems. We summarize descriptions of physical quantities as well as expressions of lumped parameters with respect to physical quantities in Table 1, for more details we refer to Yang et al. (2015).

### 2.2. Effects of single-model parameters on detection thresholds

Here, we study how the model-based detection threshold changes by sweeping values of single physical or lumped parameters. In the model, there are two kinds of parameter redundancies. One is among the physical quantities. There are three subgroups of such parameters: {*V*_*th*_, *c*_0_, *c*_1_}, {ρ, ḡ, *K*}, and {*l*, λ_*h*_}. The other kind of redundancy reflects the relation between physical quantities and six lumped parameters: *h* and α_1_; *C*_1_ and τ_1_; *C*_2_ and τ_2_; α_*h*_ and α_*L*_; σ_*h*_ and σ_*L*_; and {*l*, λ_*h*_} and λ_*L*_. Because of this redundancy, we only give reference values of lumped parameters (see Table 1) rather than physical quantities. To simulate detection thresholds, we set α_1_ = 0.125 mA, τ_1_ = 0.2 ms, τ_2_ = 45 ms, α_*L*_ = 0.00417 A/s, $\sigma_{L}=8.33\times 10^{-5}$ A/s, and λ_*L*_ = 0.01 kHz. The magnitudes of the time constants τ_1_ and τ_2_ are similar to those from Mogyoros et al. (1996), Prescott and Koninck (2002), and Weng et al. (2006). The values of the remaining four lumped parameters are chosen to produce model-based detection thresholds similar to the experimentally observed ones (Doll et al., 2016a,b). On the basis of the expressions in Table 1, it is sufficient to consider 10 physical quantities: (*V*_*th*_, *C*_1_, *G*_1_, ρ, *h*, *C*_2_, *G*_2_, α_*h*_, σ_*h*_, and λ_*h*_) to cover all parameters. Monotonicity of the detection threshold with respect to parameters may be utilized as a qualitative measure in a model validation study when an experimental technique to tune values of a specific parameter is available. To study monotonicity in the HM, we vary the values of the parameters from 60 to 170% of the reference values. In model simulations, we use four combinations of temporal stimulus properties as used in Doll et al. (2016b): (*NoP* = 1, *PW* = 0.21 ms), (*NoP* = 1, *PW* = 0.525 ms), (*NoP* = 2, *IPI* = 20 ms, *PW* = 0.525 ms), and (*NoP* = 2, *IPI* = 50 ms, *PW* = 0.525 ms). To illustrate the possible non-monotone effects of σ_*h*_ and σ_*L*_ on detection thresholds, we use another combination of temporal stimulus properties: (*NoP* = 2, *IPI* = 30 ms, *PW* = 0.525 ms).

### 2.3. Effects of temporal stimulus properties on detection thresholds

Here, we analyse the effects of temporal stimulus properties on the detection threshold, in particular the *IPI*. As we reported in Yang et al. (2015), the threshold decreases as *PW* increases because enhanced fiber activation gives more impulses to secondary neurons. For multiple pulses, the detection threshold is also lower compared to single-pulse stimuli. For double-pulse stimuli, our previous modeling study suggests that the dependence of the threshold *A*_50_ on *IPI* could be non-monotone. In the following, we derive a condition on the values of the parameters whether this relationship is monotone or not.

**Figure 2:**
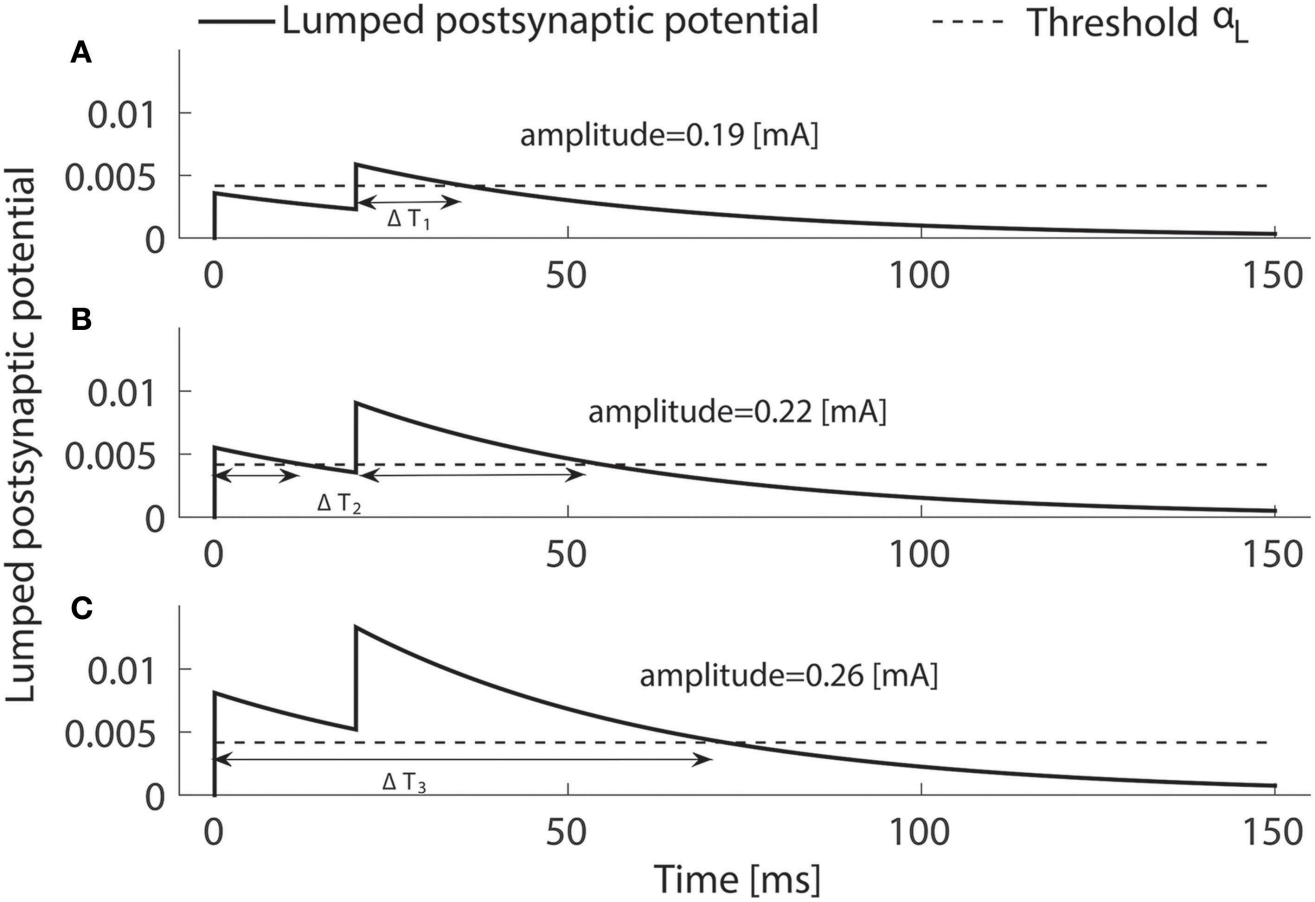
**Three ways the postsynaptic potential *x*^0^(*t*) (solid) can reach the threshold (dashed) when two pulses are applied**. The above-threshold time Δ*T* may consist of one interval by crossing upon the second pulse only **(A)** or upon the first pulse staying above threshold until the second pulses **(C)**. If the pulses are well separated in time Δ*T* consists of two intervals **(B)**. Temporal properties are *NoP* = 2, *IPI* = 20 ms and *PW* = 0.525 ms, and the amplitude are varied, i.e., not equal to *A*_50_.

For simplicity, we will restrict ourselves to the case *NoP* = 2. The effect for more pulses is similar, but less pronounced, and the analysis is more involved. First, we assume τ_*s*_ → 0, as τ_*s*_ ≪ τ_2_ based on (Gabbiani et al., 1994; Prescott and Koninck, 2002). Hence, the lumped postsynaptic potential in (2) can be simplified to

(6) $$\begin{array}{l} x^{0}\left(t\right)=B\overset{NoP-1}{\underset{k=0}{\sum }}\exp\left(-\frac{t-kIPI}{\tau_{2}}\right)H\left(t-kIPI\right), \end{array}$$

where $B=\frac{{\left[f_{A}-\alpha_{1}\right]}_{+}}{\tau_{2}}$. Second, we consider the limit σ_*h*_ → 0, resulting in σ_*L*_ → 0. This simplifies the analysis, as there is only a contribution to the integral λ_*T*_ if $x^{0}\left(t\right)>\alpha_{L}$, *t* ∈ (0, *T*). Indeed, as (3) becomes a step function, the psychometric function in (5) has the simple form Ψ = 1 − exp(−λ_*L*_Δ*T*), where Δ*T* denotes the total time *x*^0^ is above the threshold α_*L*_, see Figure 2. Now, we have three distinct cases to reach the threshold α_*L*_. First, the activity would be above threshold after the second pulse, but not the first. Second, both pulses may lead to supra-threshold activity but during two separate time intervals. Third, if a single pulse leads to supra-threshold activity, we may have one interval, if $x^{0}\left(IPI^{-}\right)>\alpha_{L}$. The time Δ*T*_*i*_, *i* = 1, 2, 3 above α_*L*_ can be calculated explicitly as:

(7) $$\begin{array}{l} \begin{array}{c} \Delta T_{1}=\tau_{2}\left(\log\left(1+\exp\left(-IPI∕\tau_{2}\right)\right)-\log\left(\frac{\alpha_{L}}{B}\right)\right),\\ \Delta T_{2}=\tau_{2}\left(\log\left(1+\exp\left(-IPI∕\tau_{2}\right)\right)-2\log\left(\frac{\alpha_{L}}{B}\right)\right),\\ \Delta T_{3}=IPI+\tau_{2}\left(\log\left(1+\exp\left(-IPI∕\tau_{2}\right)\right)-\log\left(\frac{\alpha_{L}}{B}\right)\right). \end{array} \end{array}$$

To illustrate the three cases, we use double-pulse stimuli with *IPI* = 20 ms and *PW* = 0.525 ms at amplitudes *A* = 0.19, 0.22, and 0.26 mA. We simulate the lumped postsynaptic potential of the HM using the reference values for the lumped parameters in Table 1.

We consider model-based detection thresholds in these three cases. From (5), it follows that the detection threshold *A*_50_ and *IPI* are related by the implicit equation λ_*L*_Δ*T* = log(2). If the *IPI* is large, then the time above threshold is always given by Δ*T*_2_. By decreasing *IPI*, the detection threshold *A*_50_ may change such that either Δ*T*_1_ or Δ*T*_3_ should be used. The value of *IPI*, where we switch from the second case to the first, is given by:

(8) $$\begin{array}{l} IPI_{21}=-\tau_{2}\log\left(2^{1∕\left(\tau_{2}\lambda_{L}\right)}-1\right), \end{array}$$

and to the third case by:

(9) $$\begin{array}{l} IPI_{23}=\tau_{2}\log\left(\sqrt{2^{1∕\left(\tau_{2}\lambda_{L}\right)}+\frac{1}{4}}-\frac{1}{2}\right). \end{array}$$

Now, either λ_*L*_τ_2_ > 1, and then *IPI*_21_ is negative and *IPI*_23_ is positive, or λ_*L*_τ_2_ < 1 and then *IPI*_23_ is negative and *IPI*_21_ is positive. Hence, as we decrease *IPI*, we find either case 1 or case 3, and this depends on whether λ_*L*_τ_2_ is smaller or larger than one.

It is easy to see that Δ*T*_1_ and Δ*T*_2_ are decreasing functions of *IPI*, and Δ*T*_3_ is an increasing function. It follows from the definition of the psychometric function (5) that ∂*A*_50_(*IPI*)∕∂*IPI* < 0, if both *IPI* < *IPI*_23_ and λ_*L*_τ_2_ < 1; otherwise, the derivative is positive. Hence, if λ_*L*_τ_2_ < 1, then the dependence of the detection threshold on the interpulse interval is non-monotone.

### 2.4. Neuroplasticity induced by topical capsaicin treatment and relevant modeling

We consider the observed patterns of changes in detection thresholds on five study days with 1-h capsaicin treatment over three months with 8% capsaicin treatment (Doll et al., 2016b), as illustrated in Figure 1. First, we briefly review neuroplasticity caused by topical application of capsaicin. Second, with plausible changes in related model parameters, we study the patterns of changes in model-based detection thresholds.

#### 2.4.1. Capsaicin-induced neuroplasticity

Capsaicin activates the TRPV1-expressing C fibers and some Aδ-fibers (O'Neill et al., 2012). The application of a high-dose capsaicin patch results in high levels of intracellular calcium in nerve endings leading to mitochondrial dysfunction and retraction of nerve endings (Anand and Bley, 2011). In the periphery, neuropeptides, such as substance P (SP) and calcitonin gene-related peptide (CGRP), are released from the activated peptidergic C fibers, resulting in neurogenic inflammation (Coutaux et al., 2005; Voscopoulos and Lema, 2010). This inflammation is accompanied by the release of inflammatory agents such as prostaglandin E2 and histamine. In turn, these agents may trigger intracellular signaling in nociceptors through protein kinase A and C (PKA and PKC) (Khasar et al., 1999; Malmberg, 2000). These protein kinases can further modulate the properties of tetrodotoxin-resistant (TTXr) persistent sodium channels in nerve endings, thereby shifting the activation curve of these channels to more hyperpolarized values (Gilchrist and Bosmans, 2012). Hence, the excitability of Aδ-fibers may increase due to the presence of inflammatory agents. After a 60-min application of an 8% capsaicin patch, it is common to observe application-site erythema. The erythema was reported to resolve within 1–3 days after the capsaicin patch treatment (FDA Center for Drug Evaluation and Research, 2009). This suggests that capsaicin-induced neurogenic inflammation could last up to 3 days. In the superficial dorsal horn, the central terminals of peptidergic C fibers modulate the secondary neurons via release of SP (Todd, 2010). SP can further activate intracellular signaling, e.g., PKA, PKC, and extracellular-signal-regulated kinases (ERK) in superficial dorsal horn neurons, thereby resulting in shorter and longer-lasting neuroplasticity. The increased density of AMPA receptors enhances synaptic efficacy, and the reduction of A-type potassium currents increases membrane excitability (Latremoliere and Woolf, 2009). These effects can last several hours in human experimental pain models (Latremoliere and Woolf, 2009; Sandkühler, 2009) and are considered to be transcription-independent processes. The longer-lasting central neuroplasticity was suggested to be transcription-dependent, thereby possibly explaining the persistent pain state in pathological settings (Latremoliere and Woolf, 2009).

#### 2.4.2. Simulated detection thresholds over three months

We model the capsaicin-induced effects by different perturbations of physical quantities for each study day. First, for degeneration of nerve endings and their regrowth, the intra-epidermal nerve fiber density ρ is expected to decrease initially and then to return to baseline, as also observed by Kennedy et al. (2010). In addition, as the nerve endings retract from the epidermis, their depth *h* increases (O'Neill et al., 2012). Second, we increase the membrane excitability of nerve endings by lowering the firing threshold *V*_*th*_ on Day 2. This models the capsaicin-induced neurogenic inflammation, which in normal circumstances is resolved within a couple of days. Third, increases in membrane excitability and synaptic efficacy of the superficial dorsal horn neurons can be captured by decreasing *G*_2_ and increasing ḡ, respectively. Summarizing, it is plausible that changes in the physical quantities ρ, *h*, *V*_*th*_, *G*_2_, and ḡ can account for the experimental pattern in detection thresholds.

We consider the values of the model parameters on Day 0 as baseline. We use the same values for the lumped parameters as in the parameter sweeping: α_1_ = 0.125 mA, τ_1_ = 0.2 ms, τ_2_ = 45 ms, α_*L*_ = 0.00417 A/s, $\sigma_{L}=8.33\times 10^{-5}$ A/s, and λ_*L*_ = 0.01 kHz. The perturbation patterns are presented as multiplication factors (*r*_ρ_, *r*_*h*_, *r*_*V*_*th*__, *r*_*G*_2__, and *r*_ḡ_) to the baseline values, see Table 2. Hence, the actual lumped parameter values are tuned according to the expressions given in Table 1. A previous human study with the same administration of the capsaicin patch to thighs reported decreased fiber densities on Day 7 and 84 to 21 and 80% of the baseline, respectively (Kennedy et al., 2010). Here, we set these two ratios for our model simulations. In addition, as the regeneration of fiber densities approximately obeyed a linear relationship with respect to time (Polydefkis et al., 2004), we determined the ratios of fiber densities on Day 2 and 28 by linear extrapolation and interpolation based on values for densities on Day 7 and 84 from literature. Values of other parameters are changed to capture the experimentally obtained changes in detection thresholds in a systematic way, which also agrees qualitatively with fiber retraction (O'Neill et al., 2012), changes of peripheral excitability (Coutaux et al., 2005), and plasticity of the central subsystem (Latremoliere and Woolf, 2009). We compute the detection thresholds for each combination of temporal properties on each study day. To understand how changes in all five physical quantities act together, we also compute the thresholds with either peripheral or central functional plasticity omitted, or both.

**Table 2:** **Perturbation patterns of the ratios of five physical quantities**.

**Parameters**	**Day 0**	**Day 2**	**Day 7**	**Day 28**	**Day 84**
*r*_ρ_	1	0.172	0.21	0.371	0.80
*r*_*h*_	1	1.3	1.25	1.23	1.15
*r*_*V*_*th*__	1	0.45	1	1	1
*r*_*G*_2__	1	0.92	0.9	0.96	0.98
*r*_ḡ_	1	1.832	4.608	3.234	1.4

## 3. Results

We explore effects of physiological parameters on detection thresholds by sweeping parameters. Next, we discuss the effect of the *IPI* on the detection threshold by both mathematical analysis and model simulations. Lastly, we show simulated detection thresholds on the five study days in the case study of topical capsaicin treatment. These simulations show qualitative agreement with patterns of changes of experimental thresholds.

### 3.1. Effects of single model parameters on detection thresholds

**Figure 3:**
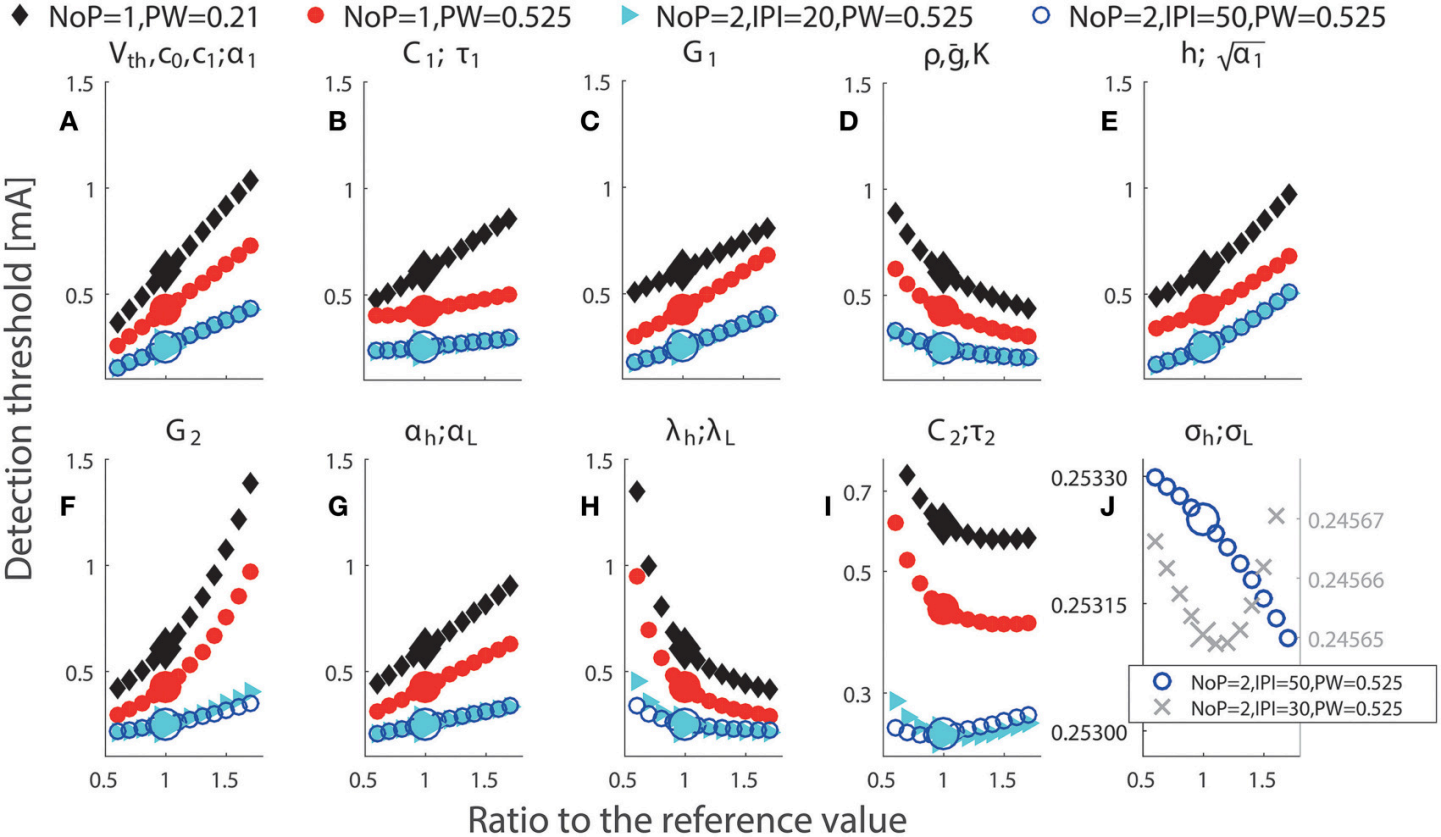
**Simulated detection thresholds with perturbation of single parameters**. The parameter changed in a particular panel is displayed above panels **(A–J)**. Black: *NoP* = 1, *PW* = 0.21 ms; Red: *NoP* = 1, *PW* = 0.525 ms; Cyan: *NoP* = 2, *IPI* = 20 ms, *PW* = 0.525 ms; Blue: *NoP* = 2, *IPI* = 50 ms, *PW* = 0.525 ms and Gray cross: *NoP* = 2, *IPI* = 30 ms, *PW* = 0.525 ms (only in **J**). The titles in panels indicate perturbed parameters. In **(I)**, the y-axis has a log-scale for better visualization of the non-monotonicity of detection thresholds with respect to *C*_2_ or τ_2_. In **(J)**, the y-axis has different scales for better visualization of the non-monotone effects of σ_*h*_ and σ_*L*_ for two combinations of temporal properties, respectively.

Each panel in Figure 3 illustrates the effect of a single model parameter on the detection threshold. Big markers show detection thresholds for the reference values in Table 1. The steeper the curve around the reference value is, the stronger the threshold depends on this parameter locally. These results show that detection thresholds can depend non-monotonically on physical quantities *C*_2_ and σ_*h*_ or lumped parameters τ_2_ or σ_*L*_. For secondary neurons, morphological properties, i.e., the thickness and the surface of the membrane, determine *C*_2_. It is not likely that *C*_2_ changes much over time, and if it would, e.g., due to cell growth, then also the membrane conductance *G*_2_ is expected to change accordingly. As $\tau_{2}=C_{2}G_{2}^{-1}$, changes in both *C*_2_ and $G_{2}^{-1}$ compensate each other, resulting in an invariant τ_2_. However, a change of the thickness of the membrane can lead to a change solely in the physical quantity *C*_2_ or the lumped parameter τ_2_. For double-pulse stimuli, we notice that two combinations for double-pulse stimuli have very similar thresholds as shown in Figure 3. We comment on this in the next subsection. For *IPI* = 30 ms and *PW* = 0.525 ms, we find a non-monotone dependence of simulated detection thresholds on σ_*h*_ or σ_*L*_, see Figure 3J. This is a small effect and may not be experimentally observable.

### 3.2. Effect of *IPI* on detection thresholds

**Figure 4:**
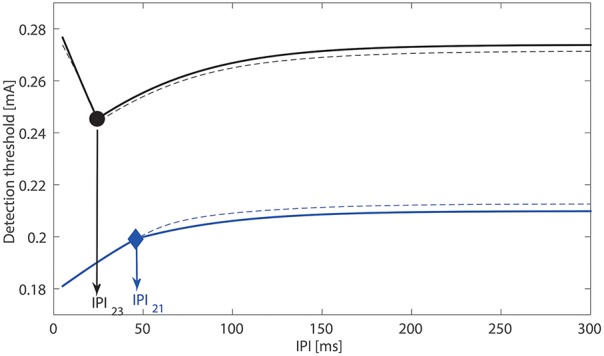
**Relationship between the detection threshold and the *IPI***. With τ_*s*_, σ_*L*_ → 0, solid curves are determined with analytically expression Δ*T*_*i*_, *i* = 1, 2, 3. The simulated detection thresholds for nonzero $\sigma_{L}:=8.33\times 10^{-5}$ A/s, τ_*s*_ = 1.5 ms are indicated by dashed curves. For the black curves, we use τ_2_ = 45 ms, λ_*L*_ = 0.01 kHz with product smaller than one. For the blue curves, we use τ_2_ = 45 ms, λ_*L*_ = 0.05 kHz with product larger than one.

Using different amplitudes of double-pulse stimuli, we illustrate the three ways how the modeled postsynaptic potential *x* can cross the activation threshold α_*L*_ of central neurons, see Figure 2. Next, as we analyzed the transition between the above different scenarios in Section 2.3, the quantity λ_*L*_τ_2_ determines the monotonicity of the detection threshold with respect to the *IPI*. Although this result is obtained in the limit when σ_*L*_ → 0 and τ_*s*_ ≪ τ_2_, we verify this also for non-zero σ_*L*_ (i.e., 8.33 × 10^−5^ A/s) and τ_*s*_ = 1.5 ms, see Figure 4. Black and blue curves depict the relationship between the detection threshold and the *IPI* with λ_*L*_ = 0.01 and 0.05 kHz, respectively, and the values of the other five lumped parameters are set to the reference values. We find that the simulated detection thresholds (dashed curves) follow the analytical expressions (plotted as solid curves) very closely. This demonstrates the validity of our analysis. As $\tau_{2}=C_{2}G_{2}^{-1}$ and λ_*L*_ = *lλ*_*h*_, changes in these four physical quantities could affect the monotonicity of detection thresholds.

### 3.3. Simulated detection thresholds and psychometric curves after topical capsaicin treatment

We have computed the model-based detection thresholds for the perturbations of five physical quantities in Table 2. We observe that there is a qualitative agreement between the simulated thresholds (red cross) and experimental data (blue) for all four combinations of temporal properties. For single-pulse stimuli, detection thresholds increase on Days 2 and 7, and for double-pulse stimuli, thresholds increase on Days 7 and 28. Moreover, for double-pulse stimuli on Day 84, the simulated detection thresholds are still larger than their baselines, which also agrees with the elevated thresholds compared to those on Day 0 (although not significant).

**Figure 5:**
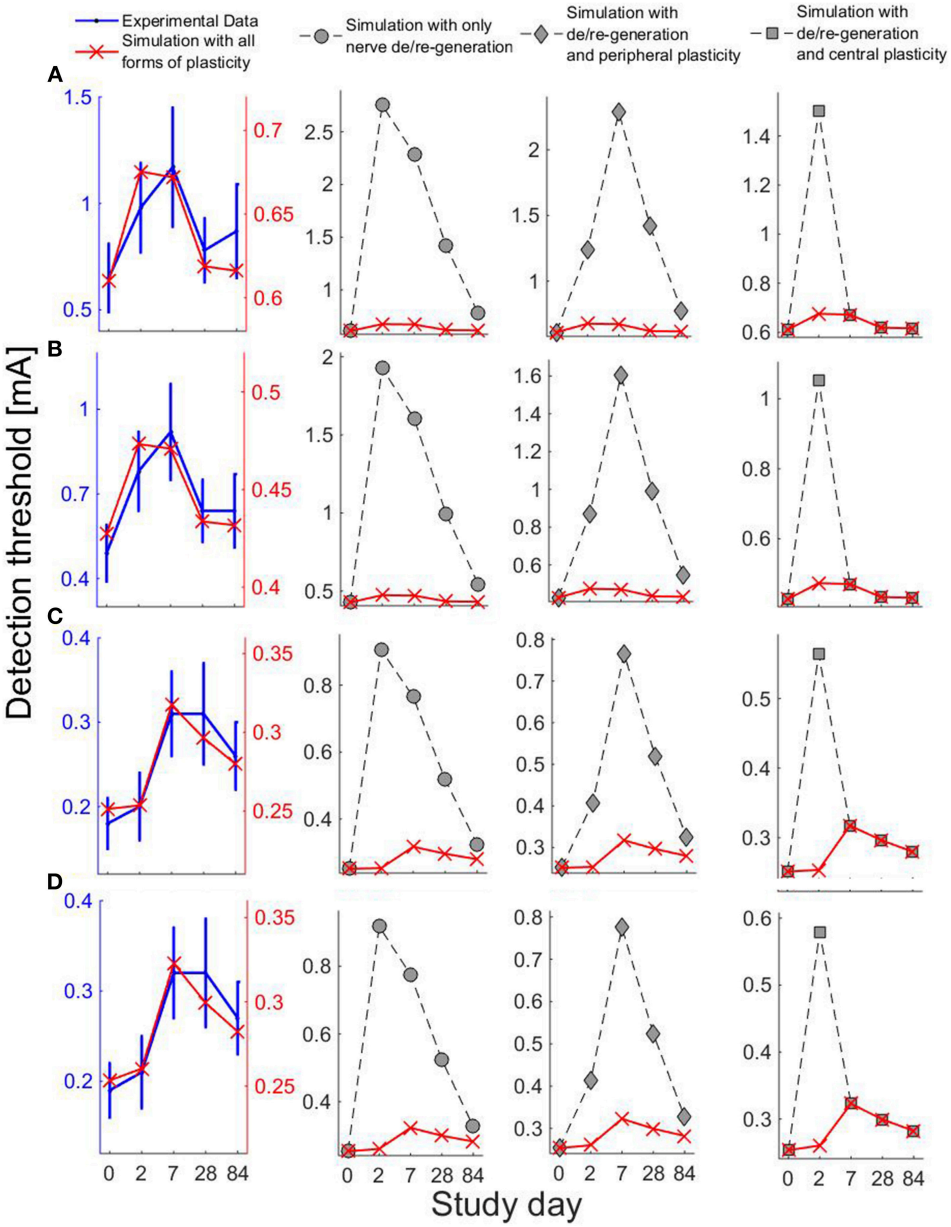
**Experiental and simulated detection thresholds on five study days using four combinations of the temporal properties**. For simulations, we consider the changes in parameters ρ, *h*, *V*_*th*_, *G*_2_, and ḡ. Row label **(A–D)** represent the four combinations of temporal properties: **(A)** (*NoP* = 1, *PW* = 0.21 ms), **(B)** (*NoP* = 1, *PW* = 0.525 ms) **(C)** (*NoP* = 2, *IPI* = 20 ms, *PW* = 0.525 ms), and **(D)** (*NoP* = 2, *IPI* = 50 ms, *PW* = 0.525 ms). The first column on the left shows simulations (red cross) with all forms of plasticity and the experimental data (blue). Other columns show simulations of detection thresholds with different combinations of forms of plasticity: circles for simulations without functional plasticity, diamonds for simulations when central functional plasticity omitted, and squares for simulations when peripheral functional plasticity omitted.

**Figure 6:**
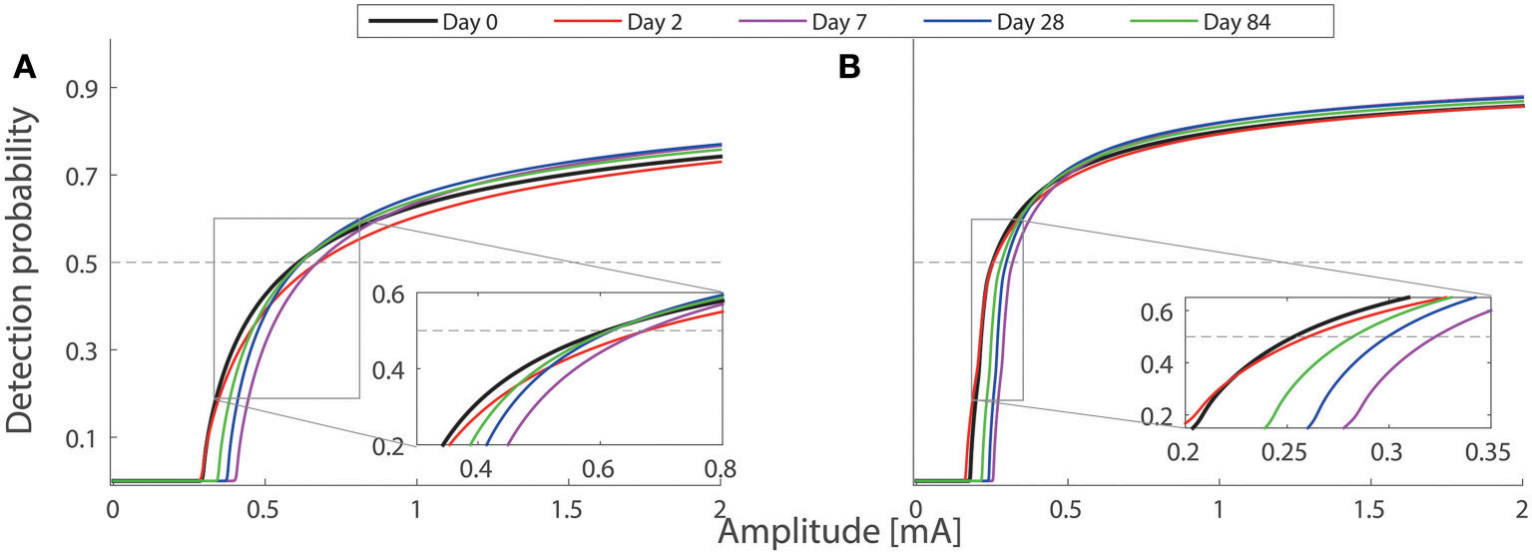
**Model-based psychometric functions on five study days**. The parameter values of the hazard model are set according to Tables 1, 2. Two combinations of temporal stimulus properties are used in the simulations: **(A)** (*NoP* = 1, *PW* = 0.21 ms) and **(B)** (*NoP* = 2, *IPI* = 20 ms, *PW* = 0.525 ms). The inset plots show two groups of psychometric functions with amplitudes near detection thresholds. The intersection between each psychometric curve and the gray horizontal line is the detection threshold. Psychometric functions for the other two combinations (*NoP* = 1, *PW* = 0.525 ms) and (*NoP* = 2, *IPI* = 50 ms, *PW* = 0.525 ms) are not shown, as they are qualitatively similar to those in **(A,B)**, respectively.

To study different combinations of neuroplasticity with the model, we omit either peripheral or central functional plasticity, or both. We present the corresponding simulated detection thresholds for these three cases in Figure 5 with gray markers. In these three cases, we find much higher thresholds. With only nerve degeneration (circles), the thresholds would increase on Days 2, 7, and 28 similarly for both single- and double-pulse stimuli. Adding only peripheral sensitization (diamonds) results in different patterns, but these simulations do not agree with detection thresholds for single-pulse stimuli observed on Day 28. With central functional plasticity and nerve degeneration, the simulation (squares) shows a qualitative disagreement for double-pulse stimuli on Day 2. We find that only all parameter changes combined lead to the same qualitative pattern as experimentally observed. The differences between thresholds for one- and two-pulse stimuli are caused by a simultaneous effect on the shape of the psychometric curve. To show, this we have computed the psychometric curves given by Equation (5) for the model on five study days for two stimulus properties (*NoP* = 1, *PW* = 0.21 ms) and (*NoP* = 2, *IPI* = 20 ms, *PW* = 0.525 ms) using the parameter values in in Tables 1, 2, see Figure 6.

## 4. Discussion

We have investigated the effects of model parameters on detection thresholds with our computational model in terms of neuroplasticity. First, we demonstrated that the detection threshold varies monotonically for most single parameters except for *C*_2_, σ_*h*_, τ_2_, and σ_*L*_ involved in central nociceptive function. Second, we developed an analytical argument why detection thresholds may depend non-monotonically on the *IPI*. Third, we translated biologically plausible capsaicin-induced neuroplasticity into perturbations in values of model parameters. Agreement between simulations and observations of detection thresholds suggests the prospective usage of this computational model for a malfunctioning nociceptive system.

### 4.1. Effects of parameters on detection thresholds

Detection thresholds depend on most of single parameters in a monotone manner, see Figure 3. In addition to obvious parameter redundancy, the effects of different parameters on detection thresholds could be similar. For example, this occurs for the pair of physical quantities *C*_1_ and α_*h*_ (equivalently for lumped parameters τ_1_ and α_*L*_) when (*NoP* = 1, *PW* = 0.21 ms), (*NoP* = 2, *IPI* = 20 ms, *PW* = 0.525 ms), or (*NoP* = 2, *IPI* = 50 ms, *PW* = 0.525 ms), see Figures 3B,G. This similarity challenges the identifiability of such model parameters, as they can compensate each other, resulting in equal detection thresholds. Hence, in future work like estimation of model parameters, we recommend to design more appropriate combinations of temporal properties to enrich the information in the data.

In a previous work (Doll et al., 2016a), varying the interpulse interval from 10 to 50 ms for double-pulse stimuli, only a small change in the detection thresholds was found. This small change could be attributed to a non-monotonic relation between *IPI* and detection thresholds (Yang et al., 2015). Our present analysis shows that the product $C_{2}G_{2}^{-1}l\lambda_{h}$ determines the monotonicity. In turn, suppose that the monotonicity can be tested in (future) experimental work, one might treat this monotonicity as an indication to infer changes of central properties *C*_2_, *G*_2_, *l*, and λ_*h*_. We acknowledge that it may be challenging to detect the (non-)monotonicity of detection thresholds with respect to the *IPI*, as statistical power may be limited for the *IPI* from 10 to 50 ms (Yang et al., 2015). For future work to verify the existence of non-monotonicity, first, one may use more or other values for the *IPI* and then study the relation between detection thresholds about these *IPI*s. Second, one could study the relation between the detection probability and the *IPI* for double-pulse stimuli by varying *IPI* but fixing *A*.

### 4.2. Modeling capsaicin-induced plasticity and its interpretation

We studied plausible neuroplasticity for the topical capsaicin treatment with the computational model. The changes of the fiber densities on study days were set partly based on literature data and also nerve regeneration with a constant regeneration rate. Other changes of physical quantities were based qualitatively on physiological mechanisms (Latremoliere and Woolf, 2009; Sandkühler, 2009; O'Neill et al., 2012). In addition, values of time constants of afferent fibers and secondary neurons were set to similar values obtained in previous experimental studies (Mogyoros et al., 1996; Prescott and Koninck, 2002; Weng et al., 2006). With these physiological restrictions on the computational model, we demonstrated qualitative agreement of simulated patterns of changes of detection thresholds to experimental patterns, see Figure 5. For a future, more quantitative study, we suggest to estimate the lumped parameters in the model using the stimulus-response pairs of single subjects. The changes in estimates of parameters might unravel the possible individual-specific nociceptive characteristics of capsaicin-induced plasticity.

Capsaicin-induced nerve degeneration is thought to underlie the therapeutic basis in pain management (Anand and Bley, 2011; O'Neill et al., 2012). Our model simulations together with observed elevations of detection thresholds on Days 2, 7, and 28 (Doll et al., 2016b) offer a theoretical support in terms of nociceptive processing. In addition, topical application of capsaicin possibly triggers two forms of neuroplasticity, thereby pushing single nociceptors or secondary neurons into sensitized states (Coutaux et al., 2005; Latremoliere and Woolf, 2009; Sandkühler, 2009). The two forms of neuroplasticity in peripheral and central nociceptive subsystems compensate the elevations in detection thresholds. Each form of plasticity results in different patterns of changes in detection thresholds for stimuli with either single- or double-pulses on the five study days. Our model simulations dissect different longitudinal effects caused by peripheral and central plasticity, as illustrated in Figure 5. Such difference provides valuable information to identify the existence of underlying plasticity.

For our model simulations on Day 2, both forms of peripheral and central plasticity are required for the small change of detection thresholds with *NoP* = 2, as well as for clear elevations of detection thresholds with *NoP* = 1. Then, the small change in detection threshold with *NoP* = 2 is less straightforwardly interpretable. Here, we provide an explanation based on how physical quantities affect psychometric curves. Nerve degeneration with a clearly decreased value of ρ and increased *h* tends to shallow Ψ and to shift Ψ to the right. However, peripheral plasticity with a decreased value of *V*_*th*_ overcomes this tendency, resulting in an effectively smaller shift of Ψ to the left. In addition, central plasticity with a decreased *G*_2_ and an increased ḡ compensates the dominant tendency with a shallower Ψ caused by the dramatic loss of nerves. In addition, it is expected that the psychometric function for double-pulse stimuli is steeper in comparison to single-pulse stimuli. Taken together, on Day 2, detection thresholds are elevated for single-pulse stimuli but not for double-pulse stimuli. The simulations of the psychometric functions are illustrated in Figure 6.

On Day 28, the modeled central neuroplasticity, especially the enhanced synaptic efficiency, compensates the loss of nerve endings, resulting in a less increased detection threshold for single-pulse stimuli but not for double-pulse stimuli. One can also explain this observation based on the distortion of psychometric functions due to changes in model parameters. As the peripheral plasticity is resolved within 3 days after capsaicin application, Ψ is expected to shift to the right due to an increased value of *h*. In addition, the central plasticity leads to an effectively steeper Ψ than the baseline as nerve endings regenerate with an increased ρ compared to that on Day 2. Hence, all these nociceptive changes result in increases of detection thresholds for double-pulse stimuli but not for single-pulse stimuli on Day 28. The simulations of the psychometric functions are illustrated in Figure 6.

### 4.3. Limitations of the model and possible extensions

Our present study uses a computational model (Yang et al., 2015) to study the effects of neuroplasticity on detection thresholds. This model is relatively simple as it assumes that an evoked spike leads to a single synaptic event at a secondary neuron. The model is mainly concerned with nerve endings in the skin and secondary neurons. There exist many models of axonal transmission, e.g., incorporating the geometry of branch points in dorsal root ganglion and distribution of myelin along the axon, leading to propagation failure and ectopic spiking behavior (Zhou and Chiu, 2001; Coggan et al., 2010, 2011; Volman and Ng, 2013, 2014), thereby possibly leading to neuropathic pain (Coggan et al., 2015). It would be interesting to incorporate the effects of axonal transmission into the hazard model to study the effect of demyelination on nociceptive detection thresholds. For the current study, however, we remark that the topical application of capsaicin is thought to focally affect free nerve endings (Anand and Bley, 2011). There has been no report, to the best of our knowledge, that this application would induce axonal injury or degeneration in myelinated axons of Aδ-fibers, which are located within deeper tissue than the dermis (Provitera et al., 2007). As we noted before, see also (Yang et al., 2015), another model extension would be to include short-term synaptic plasticity. The temporal summation of postsynaptic potentials may be affected by facilitation or depression. In turn, this would lower or increase the detection threshold, respectively. In Doll et al. (2016a), it was noted that the detection threshold for two-pulse stimuli was close to as expected based on temporal summation of postsynaptic potentials. As we model the detection as the occurrence of the first spike, supra-threshold mechanisms seem to be less relevant. Sub-threshold mechanisms could also play a role, e.g., if that changes the secondary neuron into a resonator instead of an integrator. It might be possible to study that more precisely by measuring the detection threshold for more values of *IPI*. Still, being aware of these limitations, our simple model and possible refinements might help to investigate relevant neuroplasticity induced by other medical interventions or diseases.

### 4.4. Future studies on capsaicin-induced neuroplasticity

In our case study, capsaicin-induced central plasticity appears to be present for a much longer time compared to what is usually mentioned for experimental pain models (Sandkühler, 2009). The time course of such plasticity is similar to estimated time courses found in human subject study using high frequency electrical stimulation as the conditioning stimulus (Pfau et al., 2011). Our results suggest that late-phase synaptic plasticity could have occurred in human subjects. The underlying physiological mechanisms would involve transcription-dependent processes (Kawasaki et al., 2004; Latremoliere and Woolf, 2009). Application of an 8% capsaicin patch might become a human experimental model to test possible medication to prevent or reverse the underlying hypothetical processes of this longer-lasting central plasticity. In addition, our modeling study also sheds light on the design of experiments to test the effects of certain medicine on alterations in the nociceptive system. Exploiting the capsaicin-induced plasticity in a human model, one may utilize other medical interventions together with the capsaicin treatment. One recommendation is to apply the lidocaine derivative (Binshtok et al., 2007) as well to prevent the activation of TTXr-sodium channels over a couple of days. Moreover, one could apply ketamine, an NMDA-antagonist, to effectively prevent the enhanced central plasticity (Woolf, 2011). The combination of ketamine and capsaicin would allow to further investigate contributions of peripheral and central plasticity. Accordingly, measurements with such perturbation could help to verify the presence of aforementioned abnormal spiking in axonal injuries. We recommend to compare prospective observations of detection thresholds with our model predictions of detection thresholds in Figure 5. Similar results in such a comparison would further validate our computational model. On the other hand, the possible difference from experimental observations would be useful for further refinement of the model toward more mechanistic insights of capsaicin-induced plasticity.

## Author contributions

HY, HM, RD, JB, and SV designed and conceived the study. HY and HM conducted the mathematical analysis. HY drafted the manuscript. HM, RD, JB, and SV commented and edited the manuscript. All authors read and approved the final manuscript.

### Conflict of interest statement

The authors declare that the research was conducted in the absence of any commercial or financial relationships that could be construed as a potential conflict of interest.
